# Proteomic Analysis of Kiwifruit in Response to the Postharvest Pathogen, *Botrytis cinerea*

**DOI:** 10.3389/fpls.2018.00158

**Published:** 2018-02-15

**Authors:** Jia Liu, Yuan Sui, Huizhen Chen, Yiqing Liu, Yongsheng Liu

**Affiliations:** ^1^Chongqing Key Laboratory of Economic Plant Biotechnology, Collaborative Innovation Centre of Special Plant Industry in Chongqing, College of Forestry and Life Science, Institute of Special Plants, Chongqing University of Arts and Sciences, Yongchuan, China; ^2^College of Food Science and Engineering, Hefei University of Technology, Hefei, China; ^3^College of Biology Science and Engineering, Hebei University of Economics and Business, Shijiazhuang, China

**Keywords:** defense response, gray mold, proteomics, kiwifruit-*B.cinerea* interaction, postharvest decay

## Abstract

Gray mold, caused by the fungus *Botrytis cinerea*, is the most significant postharvest disease of kiwifruit. In the present study, iTRAQ with LC-ESI-MS/MS was used to identify the kiwifruit proteins associated with the response to *B. cinerea*. A total of 2,487 proteins in kiwifruit were identified. Among them, 292 represented differentially accumulated proteins (DAPs), with 196 DAPs having increased, and 96 DAPs having decreased in accumulation in *B. cinerea*-inoculated vs. water-inoculated, control kiwifruits. DAPs were associated with penetration site reorganization, cell wall degradation, MAPK cascades, ROS signaling, and PR proteins. In order to examine the corresponding transcriptional levels of the DAPs, RT-qPCR was conducted on a subset of 9 DAPs. In addition, virus-induced gene silencing was used to examine the role of *myosin 10* in kiwifruit, a gene modulating host penetration resistance to fungal infection, in response to *B. cinerea* infection. The present study provides new insight on the understanding of the interaction between kiwifruit and *B. cinerea*.

## Introduction

Kiwifruit is subject to postharvest fungal decay, resulting in significant economic losses during storage and transport. Among postharvest diseases, gray mold, caused by the fungal pathogen *Botrytis cinerea*, is the most devastating (Park et al., 2015). Although chemical (Minas et al., 2010), physical (Chen et al., 2015), and biological (Kulakiotu et al., 2004) approaches have been developed to control gray mold of kiwifruit, a comprehensive understanding of the pathogenesis of *B. cinerea* on kiwifruit is lacking.

*B. cinerea* is a necrotrophic fungal pathogen in the Sclerotiniaceae. It has a wide host range and can infect more than 200 host plant species, being especially destructive on fruits and vegetables (Wiilliamson et al., 2007). *B. cinerea* secretes a large number of extracellular proteins that facilitate wound invasion and colonization, and thus contribute to virulence (González-Fernández et al., 2015; Liu et al., 2017). Several *B. cinerea* genes related to its growth and virulence have been characterized. Harren et al. (2012) reported that two Ca^2+^/calcineurin-dependent signaling pathway genes, *BcCnA* and *BcRcn1*, regulated fungal development and virulence in *B. cinerea*. More recently, a Rab/GTPase family gene, *Bcsas1*, was shown to impact the growth, development, and secretion of extracellular proteins in *B. cinerea*, in a manner that decreased virulence (Zhang et al., 2014).

Proteomics has emerged as a powerful tool for understanding the molecular mechanism of plant-pathogen interactions (Imam et al., 2017). Using proteomics, the response of *B. cinerea* to plant-based elicitors and hormones (Dieryckx et al., 2015; Liñeiro et al., 2016), and the *in vitro* secretome of *B. cinerea* related to pathogenesis (González-Fernández et al., 2015) have been characterized. In general, proteomic analyses of plant hosts in response to fungal pathogens have been widely reported in recent years. For instance, Zhang et al. (2017b) employed an iTRAQ-based proteomic analysis of cotton to *Rhizoctonia solani* infection and reported that ROS homeostasis, epigenetic regulation, and phenylpropanoid biosynthesis were closely associated with innate immune responses in cotton. Kumar et al. (2016) used a combined proteomic and metabolomic approach to characterize *Fusarium oxysporum* mediated metabolic reprogramming of chickpea roots. Proteomic studies of the interaction between sugarcane and *Sporisorium scitamineum* (Barnabas et al., 2016), soybean and *Fusarium virguliforme* (Iqbal et al., 2016), and ashwagandha (*Withania somnifera*) and *Alternaria alternata* (Singh et al., 2017), have also been reported. Only a couple of studies utilizing a proteomic analysis, however, have been conducted in kiwifruit shoots (Petriccione et al., 2013) and leaves (Petriccione et al., 2014) in response to the canker-causing, bacterial pathogen, *Pseudomonas syringae* pv. *actinidiae*.

In the present study, an iTRAQ-based quantitative proteomic analysis, combined with gene expression and virus-induced gene silencing (VIGS), were used to identify genes associated with the infection of kiwifruit (*Actinidia deliciosa* “Hayward”) by *B. cinerea*. To the best of our knowledge, this is the first proteomic study of the kiwifruit-*B. cinerea* interaction, and provides information that can be used to better understand the mechanism of gray mold infection in kiwifruit.

## Materials and methods

### Plant material and inoculation

**Figure 1:**
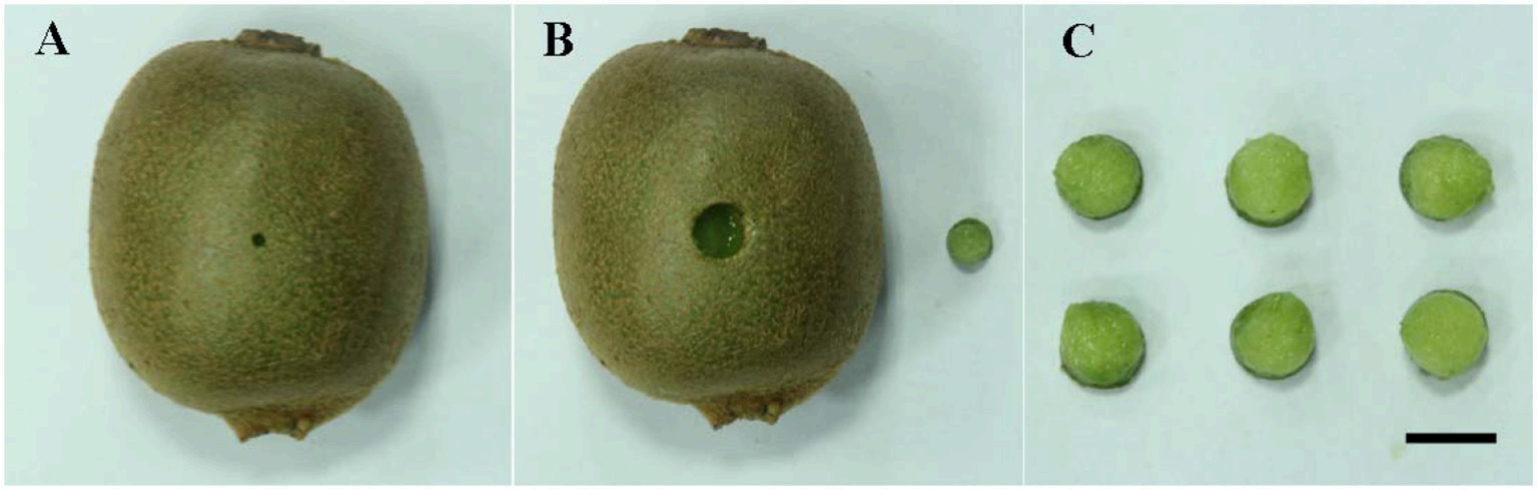
A representative picture showing the wounding and sampling of kiwifruit. **(A)** Wounded-inoculated kiwifruit prior to sampling; **(B)** Appearance of kiwifruit after sampled tissue was removed from inoculated kiwifruit; **(C)** Sampled kiwifruit tissue. Scale bar (–) represents 1 cm.

Kiwifruits (*A. deliciosa* “Hayward”) were harvested at 130 days after flowering from a research planting located in Xuancheng City, Anhui Province, China. The average quality parameters at the time of harvest were: 6.2° Brix, 56 N firmness, and 93 g fruit weight. Uniformly sized fruits, without wounds or rot, were selected and transported to the laboratory within 4 h after harvest. Fruits were then disinfected with 2% (v/v) sodium hypochlorite for 2 min, rinsed with tap water, and air-dried. *B. cinerea*, strain HFXC-16, which was originally isolated from infected kiwifruit, was grown on potato dextrose agar (PDA) for 2 weeks at 25°C (Chen et al., 2015). Two wounds (3 mm deep × 3 mm wide) were made with a sterile nail along the equator on opposite sides of each kiwifruit. Ten microliters of a *B. cinerea* spore suspension (1 × 10^4^ spores mL^−1^) or sterile water (control) were then pipetted into each wound and allowed to dry at room temperature (25°C). Wound sites were sampled after 24 h of incubation at 25°C for the proteomic analysis, using a 9-mm cork borer under aseptic conditions. The sampled tissues were immediately frozen in liquid nitrogen and stored at −80°C for subsequent proteomic analysis. A representative picture of a wounded/inoculated fruit and subsequent sampled tissue are presented in Figure 1. Each sample consisted of fruit tissue pooled from 40 wounds taken from 20 fruits. The proteomic analysis utilized three biological replicates for each treatment.

### Imaging of *B. cinerea* disease symptom development on kiwifruit

Inoculated kiwifruit tissues were collected after 24 and 36 h of incubation at 25°C and examined under a Zeiss Axioskop microscope (Carl Zeiss, Germany). Additional observations of disease symptoms caused by *B. cinerea* were made after 3 days post inoculation. Three replicates (five fruits per replicate) were examined at each time point.

### Protein preparation

Protein extraction from kiwifruit was performed as previously described (Liu et al., 2016). Kiwifruit sampled tissues were ground in liquid nitrogen. Proteins were extracted in a lysis buffer (7 M Urea, 2 M Thiourea, 4% CHAPS, 40 mM Tris-base, pH 8.5, 1 mM PMSF, and 2 mM EDTA), and sonicated on ice. The extracted proteins were reduced with 10 mM DTT at 56°C for 1 h and then alkylated by 55 mM iodoacetamide in the darkroom for 1 h. The reduced and alkylated protein mixtures were precipitated by adding 4 × volume of chilled acetone at −20°C overnight. After centrifugation at 30,000 g at 4°C, the pellet was dissolved in 0.5 M TEAB (Applied Biosystems, USA) and sonicated in ice. After centrifugation at 30,000 g at 4°C, an aliquot of the supernatant was taken for determination of protein concentration with a EZQ Protein Quantitation Kit (Invitrogen, USA). The proteins in the supernatant were kept at −80°C until further analysis.

### iTRAQ labeling and SCX fractionation

An aliquot of total protein (100 μg) was removed from each sample solution and digested with trypsin (Promega, USA) at 37°C for 16 h using a 30:1 protein/trypsin ratio. After trypsin digestion, peptides were passed through C18 desalting columns (Nest Group Inc, USA) and subsequently lyophilized to dryness. iTRAQ labeling was performed according to the manufacturer's instructions for an 8-plex kit (Applied Biosystems). Specifically, six samples (three biological replicates from non-inoculated controls and three biological replicates from *B. cinerea*-inoculated samples) were iTRAQ labeled: 114-, 117-, and 119-iTRAQ tags for three control replicates; 116-, 118-, 121-iTRAQ tags for three *B. cinerea*-inoculated replicates. The peptides were labeled with the isobaric tags and then incubated at room temperature for 2 h. The labeled peptide mixtures were then pooled and dried by vacuum centrifugation.

SCX chromatography was performed using a LC-20AB HPLC Pump system (Shimadzu, Japan), according to Luo et al. (2015). The iTRAQ-labeled peptide mixtures were reconstituted in 4 mL of buffer A (25 mM NaH_2_PO_4_ in 25% ACN, pH 2.7) and loaded onto a 4.6 × 250 mm Ultremex SCX column containing 5-μm particles (Phenomenex, USA). The peptides were eluted at a flow rate of 1 mL per min with a gradient of buffer A for 10 min, 5–60% buffer B (25 mM NaH_2_PO_4_, 1 M KCl in 25% ACN, pH 2.7) for 27 min, and 60–100% buffer B for 1 min. The system was then maintained at 100% buffer B for 1 min before equilibrating with buffer A for 10 min prior to the next injection. Elution was monitored at absorbance of 214 nm, and fractions were collected every 1 min. The eluted peptides were pooled into 20 fractions, desalted with a Strata X C18 column (Phenomenex) and lyophilized for subsequent LC-ESI-MS/MS analysis.

### LC-ESI-MS/MS analysis based on triple TOF 5600

LC-ESI-MS/MS analysis utilizing Triple TOF 5600 was conducted based on a protocol described in a previous study (Luo et al., 2015). Each fraction was resuspended in buffer A (5% ACN, 0.1% FA) and centrifuged at 20,000 g for 10 min. The final concentration of peptide was ~0.5 μg/μL. Ten micro liters of supernatant was loaded onto a 2-cm C18 trap column in a LC-20AD nano-HPLC (Shimadzu) with an auto sampler. The peptides subsequently were eluted onto a 10-cm analytical C18 column. The samples were loaded at 8 μL/min for 4 min, then a 35 min gradient was run at 300 nL/min starting from 2 to 35% buffer B (95% ACN, 0.1% FA), followed by 5 min linear gradient to 60%, followed by a 2 min linear gradient to 80%, and maintenance at 80% buffer B for 4 min, and finally returned to 5% in 1 min.

Data was acquired using an ion spray voltage of 2.5 kV, curtain gas of 30 psi, and nebulizer gas of 15 psi at an interface heater temperature of 150°C on a TripleTOF 5600 System (AB SCIEX, USA) fitted with a Nanospray III source (AB SCIEX) and a pulled quartz tip as the emitter (New Objectives, USA). The MS was operated with a RP of ≥ 30,000 FWHM for TOF MS scans. Survey scans for IDA were acquired in 250 ms, and 30 product ion scans were collected if the scans exceeded a threshold of 120 counts per second with a 2+ to 5+ charge-state. Total cycle time was set to 3.3 s. The Q2 transmission window was 100 Da for 100%. Four time bins were summed for each scan at a pulser frequency value of 11 kHz by monitoring the 40 GHz multi channel TDC detector with a four-anode channel ion detector. A sweeping collision energy setting of 35 ± 5 eV, coupled with iTRAQ adjust rolling collision energy, was applied to precursor ions for collision-induced dissociation. Dynamic exclusion was set for 1/2 of peak width (15 s), and the precursor was subsequently refreshed off the exclusion list.

### Proteomic data analysis

Raw data files acquired from Triple TOF 5600 were converted into MGF files using Proteome Discoverer 1.2 (Thermo, Germany), and the MGF files were queried. Protein identification was performed using the Mascot search engine v.2.3.02 (Matrix Science, UK) against a database derived from the Kiwifruit Genome, which includes 39,040 protein sequences (http://bioinfo.bti.cornell.edu/cgi-bin/kiwi/download.cgi).

Proteins were identified using a mass tolerance of ±0.05 Da (ppm) that was allowed for intact peptide masses and ±0.1 Da for fragmented ions, with an allowance for one missed cleavage in the trypsin digests. Gln->pyro-Glu (N-term Q), Oxidation (M), and deamidated (NQ) were selected as potential variable modifications, while carbamidomethyl (C), iTRAQ8plex (N-term), and iTRAQ8plex (K) were selected as fixed modifications. The charge states of peptides were set to +2 and +3. Specifically, an automatic decoy database search was performed in Mascot, along with a search of the real database, by choosing the decoy checkbox in which a random sequence of the database was generated and tested for raw spectra. Only peptides with significance scores (≥20) at the 99% confidence interval by a Mascot probability analysis greater than “identity” were counted as identified in order to reduce the probability of false peptide identification. Each confident protein identification required at least one unique peptide. The false discovery rate (FDR) of identified proteins was ≤ 0.01.

For protein quantization, a protein was required to contain at least two unique peptides. The quantitative protein ratios were weighted and normalized by the median ratio in Mascot. Only ratios with *P* < 0.05, according to a Student's *t*-test, were employed, and only fold-changes >1.33 were considered as significant. Functional annotation of the proteins was conducted using Blast2GO (https://www.blast2go.com/) against the NCBI non-redundant protein database. The KEGG (http://www.genome.jp/kegg/) and COG databases (http://www.ncbi.nlm.nih.gov/COG/) were used to classify the identified proteins. In order to provide clarity, a workflow diagram regarding the above experimental procedure from protein extraction to proteomic data analysis has been shown in Figure S1. The mass spectrometry proteomics data have been deposited to the ProteomeXchange Consortium via the PRIDE (Vizcaino et al., 2016) partner repository with the dataset identifier PXD008589.

### RT-qPCR analysis

Tissue samples were collected from fruit subjected to the same treatment conditions described for the proteomic analysis. Approximately 500 mg of fruit tissue from each sample was frozen and ground in liquid nitrogen. Total RNA was extracted using a Plant Total RNA Isolation Kit (Biofit Tech, China). The extracted RNA was treated with DNase, and purified using an EasyPure Plant RNA Kit (TransGen Biotech, China). First-strand cDNAs were synthesized using TransScript One-Step gDNA Removal and cDNA Synthesis SuperMix (TransGen Biotech). The resulting cDNAs were used for RT-qPCR analysis following the manufacturer's protocol. Briefly, each RT-qPCR reaction was carried out in a 20 μL reaction containing 10 μL of TransStart® Top Green PCR Master Mix (TransGen Biotech) and 0.4 μL of each PCR primer at 10 μM. The RT-qPCR was conducted on a ABI StepOne Plus (Applied Biosystems) using the following cycling conditions: 95°C for 30 s, followed by 40 cycles of 95°C for 5 s and 60°C for 20 s. Nine genes were selected for verification based on their pattern of differential expression revealed in the iTRAQ analysis. *EF1*α and *Actin* genes were used as internal controls (Nieuwenhuizen et al., 2009; Li et al., 2010), and relative expression was calculated using the 2^−ΔΔ*CT*^ method. Melting curve analyses of amplification products were performed at the end of each PCR reaction to ensure that unique products were amplified. PCR products were cloned and sequenced to verify their identity. The gene-specific primer pairs used for each gene are listed in Table S1. Each of the treatment groups consisted of three biological replicates, and the experiment was repeated three times. A Student's *t*-test was used to determine whether the relative difference between sample groups (*B. cinerea*-inoculated vs. water-inoculated, control kiwifruits) was statistically significant (*P* < 0.05).

### Vigs of *Myosin 10* in kiwifruit

VIGS of *Myosin10* was carried out as previously described (Liu et al., 2014). Kiwifruits obtained from the same collection of fruits used in the proteomic and RT-qPCR analyses were also used for the VIGS experiment. These fruits were harvested at 130 days after flowering. *Myosin 10* was PCR-amplified from kiwifruit cDNA using the primers: F, 5′-TCTAGAGAAACGAACAGAGATAAAATCAGAC-3′; R, 5′-CTCGAGCGCCTGTAAGGGACAAAAG-3′, with Xba I and XhoI sites (underlined) added to each end, respectively. The amplified PCR product was cloned into the pTRV2 vector and the resulting CaMV 35S promoter-driven constructs were subsequently introduced into *Agrobacterium tumefaciens* strain GV3101. Freshly-grown cultures of the transformed *A. tumefaciens* carrying the pTRV2 vector were mixed 1:1 with *A. tumefaciens* GV3101 carrying the pTRV1 vector. The mixed *Agrobacterium* cultures containing pTRV2:*CaMyosin10* and pTRV1 (OD_600_ of 0.8) were syringe-injected into kiwifruit. Mixed *Agrobacterium* cultures containing pTRV2 (empty vector) and pTRV1 served as a control.

Seven days after *Agrobacterium* injection, *B. cinerea* spores (10 μL containing 1 × 10^4^ spores mL^−1^) were inoculated into the same wounds as those created by the previously injected *Agrobacterium*. In order to maintain a high relative humidity (~85%), the treated kiwifruit were placed in covered plastic food trays enclosed in polyethylene bags and stored at 25°C in a programmable environmental chamber with a temperature and humidity control system (Sanyo, Japan). Disease symptoms caused by *B. cinerea* became apparent after 60 h of storage, and kiwifruit tissues were collected at that time for *Myosin 10* expression analysis. The experimental design consisted of three replicates of 10 fruits (two wounds per fruit) for each treatment. The experiment was repeated three times.

## Results and discussion

### Development of *B. cinerea* infection in kiwifruit

**Figure 2:**
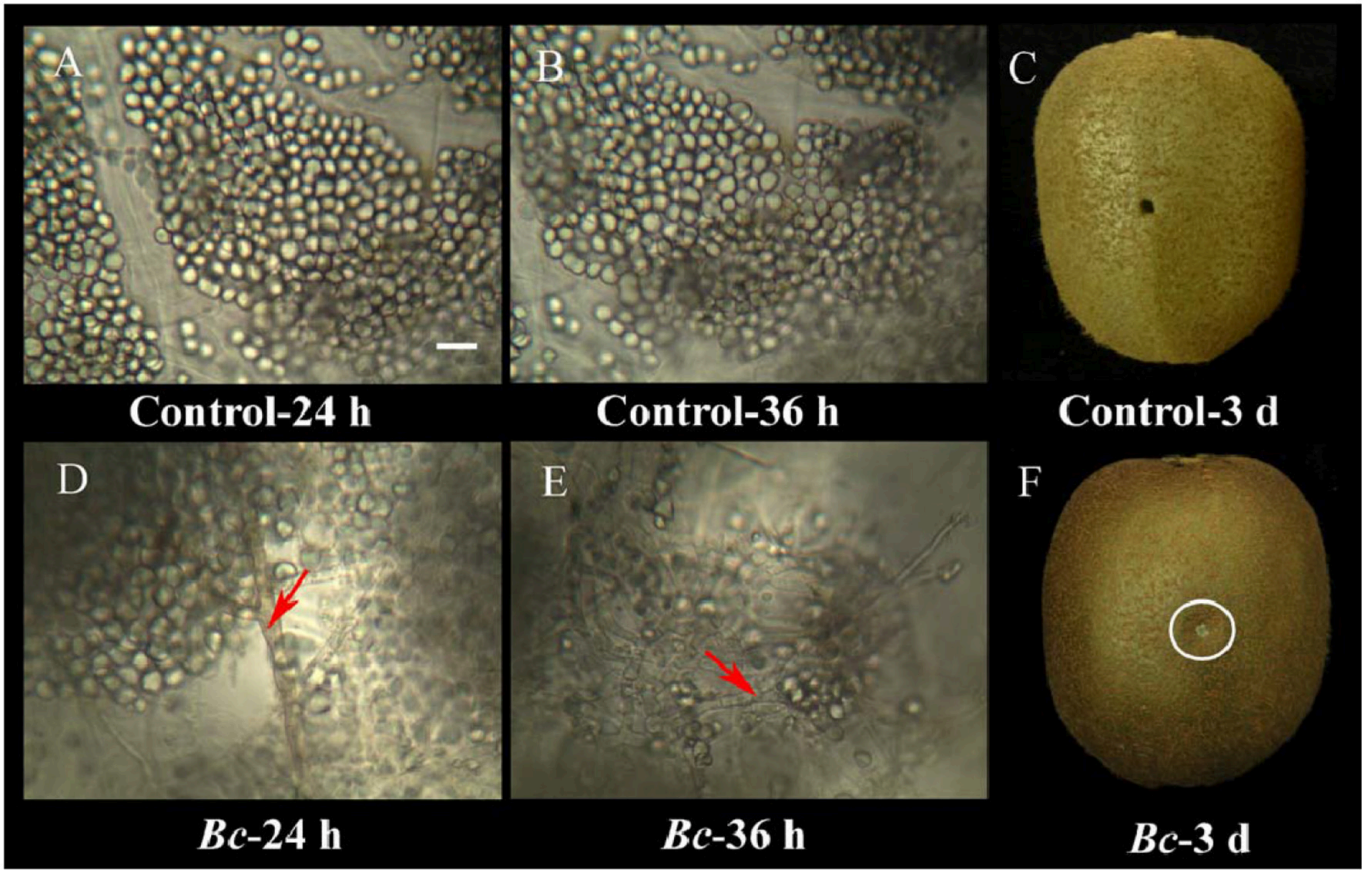
Microscopic observations of the interaction between kiwifruit and *B. cinerea* during the early stages of the infection process. Control kiwifruit tissue (inoculated with sterile water) at 24 h **(A)** and 36 h **(B)**, as well as whole fruit at 3 days post-inoculation **(C)**. Kiwifruit tissue that had been inoculated with *B. cinerea* at 24 h **(D)** and 36 h **(E)**, and whole fruit at 3-days **(F)**. Red arrows indicate *B. cinerea* hyphae. The wound inoculated with *B. cinerea* is in the area within the white circle. Scale bar (–) represents 10 μm, and is applicable to **(A–E)**.

*B. cinerea* infection of kiwifruit was clearly evident in the 3-day period of examination (Figure 2). While the kiwifruit tissue in the water-inoculated control remained intact during the 3-day storage at 25°C (Figures 2A–C), *B. cinerea* hyphae were easily observed at 24 h after inoculation in the pathogen-inoculated samples, however, the majority of the fruit cells did not appear to be degraded (Figure 2D). Based on these observations, a 24 h time point was selected for the proteomic analysis. After 24 h, fruit cells in the *B. cinerea*-inoculated samples appeared degraded, and *B. cinerea* hyphae were well established by 36 h after inoculation (Figure 2E). Macroscopic symptoms of gray mold infection of kiwifruit were readily apparent by 3 days after inoculation (Figure 2F).

### Proteomic analysis of kiwifruit in response to *B. cinerea*

Using iTRAQ and LC-ESI-MS/MS, a total of 2,487 kiwifruit proteins were identified against a database derived from the Kiwifruit Genome (http://bioinfo.bti.cornell.edu/cgi-bin/kiwi/download.cgi) (Table S2). In addition, 113 *B. cinerea* proteins were identified against a *B. cinerea* database in Uniprot (http://www.uniprot.org/uniprot/?query=%09Botryotinia+fuckeliana+&sort=score). The source should be the spores in the wound-site samples, though the amount of fungal biomass was little. The present study, however, focused on the response of kiwifruit to *B. cinerea*. The kiwifruit proteins were further investigated in the following studies.

A value of 33% fold-difference (*B. cinerea* inoculation vs. water control) was used to identify differentially accumulated proteins (DAPs) within the obtained kiwifruit protein dataset. This percentage of fold-change identified proteins that had significantly (*P* < 0.05) increased (1.33-fold) or decreased (0.75-fold) in their level of accumulation. Based upon these criteria, 196 proteins with increased and 96 proteins with decreased levels of accumulation were identified (Table 1).

**Table 1:** List of the 196 kiwifruit proteins that exhibited an increase in their level of accumulation in response to infection by *B. cinerea*, and the 96 proteins that decrease in their level of accumulation in response to infection.

**No**.	**Hits**	**Accession**	**Description**	**Fold change (mean ± SD)**
1	2277	Achn064441	Pectinesterase	4.02 ± 0.27
2	1148	Achn007441	Putative 60S ribosomal protein L35	3.54 ± 0.47
3	2317	Achn241831	UDP-glycosyltransferase 1	2.78 ± 0.73
4	557	Achn188281	Late embryogenesis abundant hydroxyproline-rich glycoprotein	2.64 ± 0.57
5	2092	Achn254861	Proactivator polypeptide	2.61 ± 0.20
6	867	Achn356861	Oxygen-evolving enhancer protein 3-2	2.56 ± 0.11
7	793	Achn024621	Epoxide hydrolase 2	2.43 ± 0.17
8	277	Achn126481	Polygalacturonase-inhibitor protein	2.43 ± 0.39
9	1909	Achn129791	40S ribosomal protein S21	2.42 ± 0.91
10	1139	Achn011001	Pectinesterase	2.30 ± 0.05
11	2331	Achn064451	Pectinesterase	2.28 ± 0.43
12	2344	Achn012841	60S ribosomal protein L26	2.23 ± 0.46
13	2356	Achn370161	60S ribosomal protein L3; putative	2.21 ± 0.04
14	2126	Achn228701	Acyl-CoA binding protein 6	2.18 ± 0.60
15	2300	Achn350811	60S ribosomal protein L17	2.14 ± 0.11
16	1221	Achn183331	60S ribosomal protein L21	2.13 ± 0.18
17	20	Achn061151	Charged multivesicular body protein 4b; putative	2.11 ± 0.68
18	1683	Achn384861	Inositol monophosphatase family protein	2.05 ± 0.96
19	1197	Achn174791	60S ribosomal protein L17	2.03 ± 0.13
20	1836	Achn244961	Putative polyvinylalcohol dehydrogenase	2.02 ± 0.19
21	2000	Achn331551	Myosin-11	2.01 ± 0.39
22	223	Achn163511	Proton pump interactor 1	1.98 ± 0.18
23	130	Achn153551	30S ribosomal protein S12; related	1.96 ± 0.16
24	2084	Achn008021	60S ribosomal protein L23a; putative	1.96 ± 0.05
25	760	Achn065911	40S ribosomal protein S11; putative	1.94 ± 0.61
26	616	Achn304291	50S ribosomal protein L2	1.94 ± 0.29
27	1920	Achn170451	Methionine aminopeptidase	1.92 ± 0.60
28	1382	Achn007231	At2g31160/T16B12.3	1.91 ± 0.27
29	1574	Achn007361	Histone H4	1.90 ± 0.24
30	1274	Achn159241	Subtilisin-like protease	1.89 ± 0.16
31	2202	Achn223851	Cyclin-dependent kinase A	1.87 ± 0.20
32	213	Achn058851	Subtilisin-like protease	1.86 ± 0.49
33	940	Achn278601	Reticulon family protein	1.86 ± 0.30
34	1520	Achn032271	Ubiquitin/ribosomal protein S27a	1.85 ± 0.57
35	826	Achn228711	Ubiquinol oxidase	1.84 ± 0.18
36	1557	Achn081501	Remorin; putative	1.82 ± 0.07
37	1597	Achn127311	Small ubiquitin-related modifier 2	1.82 ± 0.23
38	362	Achn092681	Hsc70-interacting protein	1.82 ± 0.27
39	1422	Achn128371	60S ribosomal protein L3; putative	1.81 ± 0.36
40	2343	Achn191291	40S ribosomal protein S26; putative	1.81 ± 0.29
41	137	Achn291371	Leucine-rich repeat receptor-like serine/threonine-protein kinase	1.80 ± 0.26
42	255	Achn337171	Mitochondrial import inner membrane translocase subunit tim9	1.79 ± 0.18
43	956	Achn304031	Cytochrome P450; putative	1.79 ± 0.41
44	373	Achn269851	Putative serine carboxypeptidase	1.78 ± 0.11
45	1368	Achn190951	Adenosylhomocysteinase	1.78 ± 0.68
46	517	Achn331491	Reticulon family protein	1.77 ± 0.08
47	1721	Achn132881	Myosin-10	1.77 ± 0.04
48	2001	Achn330021	Prefoldin subunit; putative	1.77 ± 0.24
49	1174	Achn155131	Syntaxin	1.77 ± 0.53
50	2351	Achn052551	V-type proton ATPase subunit G 1	1.76 ± 0.22
51	1798	Achn026511	Ribosomal protein L15	1.75 ± 0.14
52	2458	Achn358621	Heavy-metal-associated domain-containing protein; putative; expressed	1.74 ± 0.12
53	2354	Achn089541	Stress-induced-phosphoprotein	1.73 ± 0.11
54	1097	Achn001561	Stress-induced-phosphoprotein	1.72 ± 0.29
55	257	Achn151071	Adenosylhomocysteinase	1.72 ± 0.63
56	45	Achn058601	Protein grpE; putative	1.71 ± 0.03
57	782	Achn343961	Dehydrin 2	1.70 ± 0.51
58	941	Achn147681	Ly 5~-AMP-activated protein kinase beta-1 subunit-related	1.70 ± 0.41
59	1458	Achn348701	Lysosomal alpha-mannosidase; putative	1.69 ± 0.27
60	1760	Achn149381	Harpin inducing protein	1.69 ± 0.45
61	237	Achn290561	60S ribosomal protein L3; putative	1.68 ± 0.14
62	1783	Achn183021	Putative regulator of chromosome condensation; 48393-44372	1.68 ± 0.45
63	1809	Achn323431	Kinase family protein	1.68 ± 0.39
64	335	Achn281881	Putative subtilisin-like protease	1.67 ± 0.07
65	1949	Achn246321	Polygalacturonase-inhibitor protein	1.67 ± 0.11
66	175	Achn231901	60S ribosomal protein L18a	1.65 ± 0.26
67	2413	Achn105821	Calcium-binding EF hand family protein	1.65 ± 0.17
68	1398	Achn112171	RNA polymerase II C-terminal domain phosphatase-like protein	1.64 ± 0.38
69	615	Achn293101	Guanine nucleotide exchange factor	1.64 ± 0.38
70	778	Achn135031	Serine carboxypeptidase; putative	1.64 ± 0.25
71	1695	Achn036091	60S ribosomal protein L35a	1.64 ± 0.15
72	1526	Achn153791	Phenylalanine ammonia-lyase	1.64 ± 0.17
73	1496	Achn124041	30S ribosomal protein S5	1.63 ± 0.28
74	1553	Achn216701	60S ribosomal protein L7; putative	1.62 ± 0.18
75	1989	Achn011841	Late embryogenesis abundant hydroxyproline-rich glycoprotein	1.62 ± 0.24
76	1934	Achn386391	Ribosomal protein L19	1.60 ± 0.35
77	1094	Achn250781	40S ribosomal protein S13; putative	1.59 ± 0.41
78	879	Achn078681	60S ribosomal protein L13a; putative	1.59 ± 0.19
79	1013	Achn107321	Pectinesterase-2; putative	1.58 ± 0.27
80	518	Achn144051	Glutathione S-transferase 1	1.58 ± 0.24
81	2187	Achn020161	Laccase-like protein	1.58 ± 0.36
82	2475	Achn048361	Serine-threonine protein kinase	1.58 ± 0.39
83	1901	Achn074971	Pectin acetylesterase	1.57 ± 0.41
84	127	Achn363441	Lysosomal Pro-X carboxypeptidase	1.57 ± 0.21
85	1357	Achn261051	Dynamin-2B	1.57 ± 0.29
86	1138	Achn178831	Translocon-associated protein; alpha subunit; putative	1.56 ± 0.26
87	1647	Achn312631	Aldehyde dehydrogenase; putative	1.55 ± 0.16
88	1839	Achn038071	Cytochrome P450; putative	1.54 ± 0.52
89	1393	Achn226071	60S ribosomal protein L7; putative	1.54 ± 0.24
90	2384	Achn083081	50S ribosomal protein L2	1.53 ± 0.46
91	2314	Achn054521	Unknown protein	1.53 ± 0.52
92	41	Achn051951	Mitochondrial carrier-like protein	1.53 ± 0.44
93	360	Achn349511	NADH oxidoreductase F subunit	1.52 ± 0.19
94	1433	Achn228601	WD-repeat protein; putative	1.52 ± 0.41
95	47	Achn180221	Heat stress transcription factor A-5	1.52 ± 0.50
96	885	Achn178681	Ammonium transporter	1.52 ± 0.56
97	1099	Achn198781	Myosin-like protein	1.51 ± 0.56
98	1081	Achn118801	Senescence-associated protein	1.51 ± 0.19
99	2411	Achn216951	Histidine-tRNA ligase	1.51 ± 0.41
100	379	Achn061701	Cathepsin B-like cysteine proteinase 1	1.50 ± 0.06
101	1529	Achn180381	Bromodomain protein	1.50 ± 0.52
102	1751	Achn043281	Transferase; transferring glycosyl groups; putative	1.49 ± 0.28
103	2151	Achn097151	Protein phosphatase 2c; putative	1.49 ± 0.37
104	76	Achn374871	Tetratricopeptide repeat-containing protein (Precursor)	1.49 ± 0.27
105	1999	Achn074221	60S ribosomal protein L27A	1.49 ± 0.17
106	1795	Achn151811	Photosystem II protein Psb27	1.49 ± 0.17
107	1607	Achn174421	Elongation factor 1 beta	1.49 ± 0.22
108	1854	Achn127771	Mitochondrial import receptor subunit TOM9-2	1.48 ± 0.40
109	92	Achn079561	Heat shock protein 90-2	1.48 ± 0.27
110	153	Achn199371	Phospholipid-transporting ATPase; putative	1.48 ± 0.15
111	326	Achn078621	Pantothenate synthetase	1.48 ± 0.45
112	139	Achn349381	Anthranilate N-benzoyltransferase protein; putative	1.47 ± 0.15
113	423	Achn225821	ABI3-interacting protein 2	1.47 ± 0.23
114	1654	Achn151591	CASP-like protein	1.47 ± 0.12
115	1299	Achn019431	Aquaporin	1.46 ± 0.18
116	1168	Achn313721	Purple acid phosphatase 1	1.46 ± 0.28
117	586	Achn112731	Serine carboxypeptidase; putative	1.46 ± 0.27
118	995	Achn048881	Eukaryotic translation initiation factor; putative	1.46 ± 0.05
119	691	Achn121661	ATP-binding cassette transporter 1	1.46 ± 0.20
120	9	Achn197261	Proteasome subunit alpha type	1.46 ± 0.14
121	1804	Achn094391	Developmentally regulated GTP-binding protein; putative	1.46 ± 0.49
122	2188	Achn074681	Cytochrome c; putative	1.46 ± 0.19
123	2107	Achn085281	Dihydropyrimidinase; putative	1.45 ± 0.22
124	198	Achn388771	WD-40 repeat-containing protein	1.45 ± 0.33
125	1750	Achn332471	Myosin-10	1.45 ± 0.08
126	1341	Achn146501	Metacaspase 1	1.45 ± 0.17
127	2396	Achn252451	Outer envelope pore protein 37; chloroplastic	1.45 ± 0.43
128	356	Achn039991	60S ribosomal protein L5	1.45 ± 0.06
129	1416	Achn274341	60S ribosomal protein L22-like protein	1.45 ± 0.10
130	2007	Achn361381	Calcineurin B-like protein 2	1.45 ± 0.12
131	1972	Achn022101	Amine oxidase	1.44 ± 0.37
132	1775	Achn274801	60S ribosomal protein L13	1.43 ± 0.29
133	55	Achn261991	3-hydroxyacyl-[acyl-carrier-protein] dehydratase FabZ	1.42 ± 0.24
134	1005	Achn186181	RING-H2 finger protein RHF2a; putative; expressed	1.42 ± 0.55
135	663	Achn345701	50S ribosomal protein L5	1.42 ± 0.15
136	1580	Achn334211	Probable potassium transport system protein kup	1.42 ± 0.23
137	1507	Achn082021	Protein disulfide isomerase; putative	1.42 ± 0.39
138	2474	Achn288981	NADH dehydrogenase 1 alpha subcomplex subunit 13	1.42 ± 0.32
139	755	Achn314741	Cytochrome P450	1.42 ± 0.30
140	402	Achn389291	Ras-related protein Rab-2-A	1.41 ± 0.17
141	1743	Achn132141	T-complex protein 1 subunit beta	1.41 ± 0.30
142	613	Achn246001	Nascent polypeptide-associated complex subunit alpha-like protein	1.41 ± 0.15
143	436	Achn034101	LETM1 and EF-hand domain-containing protein 1; mitochondrial	1.41 ± 0.27
144	2171	Achn011061	Exocyst complex protein EXO70	1.41 ± 0.26
145	2042	Achn281431	Polyadenylate-binding protein; putative	1.41 ± 0.33
146	415	Achn006331	Cathepsin B-like cysteine proteinase 1	1.40 ± 0.34
147	413	Achn017571	Phosphoesterase family protein	1.40 ± 0.10
148	758	Achn107611	60S ribosomal protein L12; putative	1.40 ± 0.20
149	2282	Achn214241	U1 small nuclear ribonucleoprotein A	1.40 ± 0.15
150	619	Achn116721	Soul heme-binding family protein	1.40 ± 0.32
151	728	Achn068571	Ribosomal protein	1.39 ± 0.18
152	491	Achn032901	60S ribosomal protein L6	1.39 ± 0.22
153	1957	Achn198661	Developmentally regulated GTP binding protein	1.39 ± 0.22
154	1413	Achn106831	ATP-dependent Clp protease proteolytic subunit	1.39 ± 0.53
155	94	Achn383281	17.6 kDa class II heat shock protein	1.39 ± 0.41
156	418	Achn311841	Putative Molybdopterin binding; CinA-related	1.39 ± 0.06
157	585	Achn089941	DS synthase	1.38 ± 0.07
158	1082	Achn294771	Coatomer alpha subunit; putative	1.38 ± 0.38
159	1573	Achn106461	Xyloglucan-specific endoglucanase inhibitor protein	1.38 ± 0.17
160	2311	Achn341571	Calcium-binding protein; putative	1.38 ± 0.06
161	1004	Achn306081	Trigger factor; putative	1.38 ± 0.29
162	1747	Achn081801	ATP synthase D chain; mitochondrial; putative	1.38 ± 0.06
163	1324	Achn191071	Beta-galactosidase	1.37 ± 0.14
164	1484	Achn076861	Pre-mRNA-splicing factor CDC5/CEF1	1.37 ± 0.29
165	228	Achn047911	Alpha-glucosidase	1.37 ± 0.21
166	2064	Achn373051	Putative glycine-rich RNA binding protein-like	1.37 ± 0.06
167	1070	Achn132631	Thaumatin-like protein	1.37 ± 0.13
168	2432	Achn175401	Importin subunit alpha	1.37 ± 0.38
169	951	Achn073761	Reductase 2	1.37 ± 0.23
170	2303	Achn106551	Alpha-glucosidase; putative	1.37 ± 0.03
171	1635	Achn368611	FAD-binding domain-containing protein	1.36 ± 0.23
172	847	Achn022471	Kiwellin	1.36 ± 0.07
173	133	Achn191551	60S ribosomal protein L10; putative	1.36 ± 0.20
174	421	Achn314841	Proteasome subunit beta type	1.36 ± 0.32
175	316	Achn011721	Chaperone protein HtpG	1.36 ± 0.17
176	2355	Achn117921	U-box domain-containing protein 4	1.36 ± 0.18
177	935	Achn099221	Myosin-11	1.36 ± 0.18
178	674	Achn178911	Cold shock protein-1	1.35 ± 0.31
179	419	Achn202631	Protein disulfide isomerase L-2	1.35 ± 0.08
180	813	Achn087251	14-3-3-like protein GF14 Epsilon	1.35 ± 0.10
181	1117	Achn036141	Acetyl-coenzyme A carboxylase carboxyl transferase subunit alpha	1.35 ± 0.32
182	1796	Achn105661	Malic enzyme	1.35 ± 0.33
183	1204	Achn249061	HEAT repeat-containing protein 7A	1.34 ± 0.11
184	1604	Achn321291	Photosystem II D2 protein	1.34 ± 0.20
185	1383	Achn355261	Cathepsin L-like cysteine proteinase	1.34 ± 0.14
186	1944	Achn285271	Lactoylglutathione lyase; putative	1.34 ± 0.28
187	2137	Achn386611	Galactokinase; putative	1.34 ± 0.18
188	665	Achn300151	Arginine/serine-rich splicing factor; putative	1.34 ± 0.11
189	1119	Achn085181	Cop9 signalosome complex subunit; putative	1.34 ± 0.41
190	337	Achn115381	Myosin-like protein	1.33 ± 0.15
191	834	Achn071381	Chaperone protein htpG family protein	1.33 ± 0.17
192	336	Achn368931	Cytochrome P450	1.33 ± 0.36
193	1579	Achn358641	Remorin; putative	1.33 ± 0.07
194	1969	Achn353791	60S ribosomal protein L7a; putative	1.33 ± 0.03
195	267	Achn061131	Hydrogen-transporting ATP synthase; rotational mechanism; putative	1.33 ± 0.21
196	1464	Achn053521	Major latex-like protein	1.33 ± 0.07
				
197	179	Achn042071	Trafficking protein particle complex subunit	0.75 ± 0.07
198	26	Achn087361	Endoplasmic reticulum-Golgi intermediate compartment protein; putative	0.75 ± 0.12
199	2330	Achn309541	Calcineurin B subunit; putative	0.75 ± 0.06
200	2443	Achn166171	Aquaporin protein 4	0.75 ± 0.03
201	822	Achn314971	4-hydroxy-tetrahydrodipicolinate synthase	0.75 ± 0.11
202	562	Achn133811	Protein transport protein Sec61 subunit alpha	0.75 ± 0.16
203	2143	Achn185021	Mitochondrial outer membrane protein porin	0.75 ± 0.08
204	303	Achn063231	Choline-phosphate cytidylyltransferase	0.75 ± 0.10
205	2393	Achn249721	Glutamate dehydrogenase	0.74 ± 0.05
206	1069	Achn288091	Prohibitin	0.74 ± 0.22
207	1669	Achn283331	UDP-glucosyltransferase; putative	0.74 ± 0.07
208	1151	Achn162311	Reductase 1	0.74 ± 0.19
209	54	Achn230831	Wound/stress protein	0.74 ± 0.19
210	556	Achn196701	4-coumarate CoA ligase	0.74 ± 0.20
211	1589	Achn303631	Ran-binding protein 1	0.74 ± 0.17
212	1590	Achn269171	Probable UDP-arabinopyranose mutase 5	0.74 ± 0.08
213	1831	Achn235831	Beta-glucosidase	0.74 ± 0.12
214	862	Achn170351	Nudix hydrolase	0.73 ± 0.12
215	1178	Achn194491	N-carbamoyl-L-amino acid hydrolase (L-carbamoylase)	0.73 ± 0.04
216	2262	Achn285991	Glutathione peroxidase	0.73 ± 0.05
217	1241	Achn069551	Arginine–tRNA ligase	0.73 ± 0.16
218	1329	Achn193181	T-complex protein 1 subunit zeta	0.73 ± 0.05
219	535	Achn324111	Glycine cleavage system h protein; putative	0.73 ± 0.07
220	545	Achn065851	Cysteine-tRNA ligase	0.73 ± 0.21
221	970	Achn369161	Proteasome subunit beta type	0.73 ± 0.21
222	1832	Achn095061	Dolichyl-diphosphooligosaccharide-protein glycosyltransferase subunit	0.73 ± 0.08
223	1628	Achn313711	Annexin	0.73 ± 0.06
224	645	Achn311291	Glutamine-tRNA ligase; contains IPR000924 (Glutamyl/glutaminyl-tRNA synthetase; class Ib); IPR00763	0.73 ± 0.16
225	2339	Achn276041	Cystathionine beta-lyase	0.73 ± 0.14
226	1887	Achn317471	Pectinesterase inhibitor	0.73 ± 0.04
227	777	Achn122461	Aldehyde dehydrogenase	0.73 ± 0.10
228	2174	Achn022881	Proteasome subunit beta type	0.72 ± 0.11
229	1938	Achn296481	Sulfurtransferase	0.72 ± 0.25
230	1684	Achn161931	UDP-glucose 6-dehydrogenase	0.72 ± 0.01
231	2198	Achn284371	Putative delta subunit of ATP synthase	0.72 ± 0.04
232	874	Achn283441	Cyclase-like protein	0.72 ± 0.16
233	2108	Achn016261	Adenylosuccinate synthetase	0.71 ± 0.07
234	37	Achn001821	Thaumatin-like protein	0.71 ± 0.10
235	681	Achn047661	Putative RNA-binding protein	0.71 ± 0.17
236	766	Achn254211	Endoplasmic reticulum-Golgi intermediate compartment protein; putative	0.71 ± 0.13
237	1289	Achn339141	Malate dehydrogenase	0.71 ± 0.06
238	308	Achn052701	Superoxide dismutase [Cu-Zn]	0.71 ± 0.09
239	2377	Achn358201	Arginine–tRNA ligase	0.71 ± 0.07
240	74	Achn280061	Alcohol dehydrogenase; zinc-containing	0.71 ± 0.07
241	151	Achn006921	mRNA-decapping enzyme 2	0.71 ± 0.11
242	1821	Achn230841	Wound/stress protein	0.71 ± 0.01
243	795	Achn237571	Dihydroxy-acid dehydratase; putative	0.71 ± 0.09
244	1093	Achn305831	Phosphoglycerate kinase	0.71 ± 0.14
245	865	Achn227161	Patatin-like protein 3	0.70 ± 0.10
246	725	Achn364961	Glyceraldehyde-3-phosphate dehydrogenase	0.70 ± 0.11
247	2284	Achn147891	Cysteine desulfurase	0.70 ± 0.20
248	476	Achn073781	Alpha-glucan water dikinase	0.70 ± 0.06
249	1060	Achn008501	ADP-ribosylation factor	0.69 ± 0.09
250	1215	Achn147711	Oligopeptidase A; putative	0.69 ± 0.13
251	1298	Achn239461	Pyruvate kinase	0.69 ± 0.26
252	2371	Achn034821	Cytochrome P450; putative	0.69 ± 0.12
253	1866	Achn061751	Glucose-1-phosphate adenylyltransferase	0.69 ± 0.06
254	1519	Achn019301	Non-imprinted in Prader-Willi/Angelman syndrome region protein; putative	0.69 ± 0.03
255	2352	Achn349661	Glucose-6-phosphate 1-dehydrogenase	0.69 ± 0.14
256	499	Achn184951	Aspartokinase-homoserine dehydrogenase	0.68 ± 0.06
257	437	Achn077201	Glycogenin; putative	0.67 ± 0.24
258	192	Achn276181	Putative ferredoxin-dependent glutamate synthase 1	0.67 ± 0.06
259	1135	Achn268151	Acyl-CoA thioesterase; putative	0.67 ± 0.19
260	1730	Achn191941	Tryptophan synthase alpha chain	0.67 ± 0.04
261	1716	Achn146961	Proline iminopeptidase	0.66 ± 0.11
262	1488	Achn193791	Phosphate transporter	0.66 ± 0.15
263	2102	Achn042701	Protein trichome birefringence-like 38	0.66 ± 0.02
264	2463	Achn355751	Ankyrin repeat-containing protein; putative	0.66 ± 0.07
265	2378	Achn053831	Probable potassium transport system protein kup	0.66 ± 0.09
266	1455	Achn141311	Anthranilate synthase component I; putative	0.66 ± 0.17
267	1001	Achn005321	ER membrane protein complex subunit 8/9 homolog	0.66 ± 0.13
268	235	Achn109151	Inorganic pyrophosphatase protein	0.65 ± 0.06
269	2039	Achn327521	Phosphoenolpyruvate carboxylase; putative	0.65 ± 0.05
270	397	Achn123921	Polyadenylate-binding protein 1	0.65 ± 0.13
271	510	Achn259181	Putative glutathione S-transferase	0.65 ± 0.01
272	1002	Achn339791	Pentatricopeptide repeat-containing protein	0.65 ± 0.29
273	1201	Achn288731	ATP phosphoribosyltransferase	0.64 ± 0.12
274	2415	Achn114051	Glyceraldehyde-3-phosphate dehydrogenase	0.64 ± 0.13
275	1387	Achn367241	Citrate synthase	0.64 ± 0.14
276	2025	Achn001301	Putative enoyl-CoA hydratase	0.64 ± 0.11
277	1598	Achn340821	Peptidyl-prolyl cis-trans isomerase	0.63 ± 0.04
278	97	Achn387811	GRAM-containing/ABA-responsive protein	0.63 ± 0.12
279	53	Achn091801	Hydrolase; alpha/beta fold family protein	0.61 ± 0.03
280	7	Achn365261	Putative 3-oxoacyl-(Acyl-carrier protein) reductase	0.59 ± 0.09
281	509	Achn136801	26S proteasome non-ATPase regulatory subunit	0.59 ± 0.11
282	743	Achn334581	Malate dehydrogenase	0.58 ± 0.08
283	954	Achn163691	Thioredoxin	0.57 ± 0.17
284	318	Achn310551	Glyceraldehyde-3-phosphate dehydrogenase B	0.57 ± 0.18
285	1717	Achn107521	Kiwellin	0.56 ± 0.11
286	496	Achn248641	4-nitrophenylphosphatase; putative	0.55 ± 0.16
287	2016	Achn130531	Pyrophosphate-energized proton pump 1	0.54 ± 0.17
288	666	Achn350451	Glyceraldehyde-3-phosphate dehydrogenase	0.53 ± 0.07
289	1539	Achn361411	Photosystem I reaction center subunit III	0.50 ± 0.06
290	2402	Achn040571	PRA1 family protein A1	0.49 ± 0.08
291	2340	Achn331061	Germin-like protein 6	0.46 ± 0.08
292	1014	Achn236041	Putative Fatty acid oxidation complex subunit alpha	0.45 ± 0.06

*A cut-off of a 1.33 fold change in accumulation (B. cinerea inoculation vs. water control) was used to define significance (P < 0.05)*.

### Gene ontology enrichment analysis

**Figure 3:**
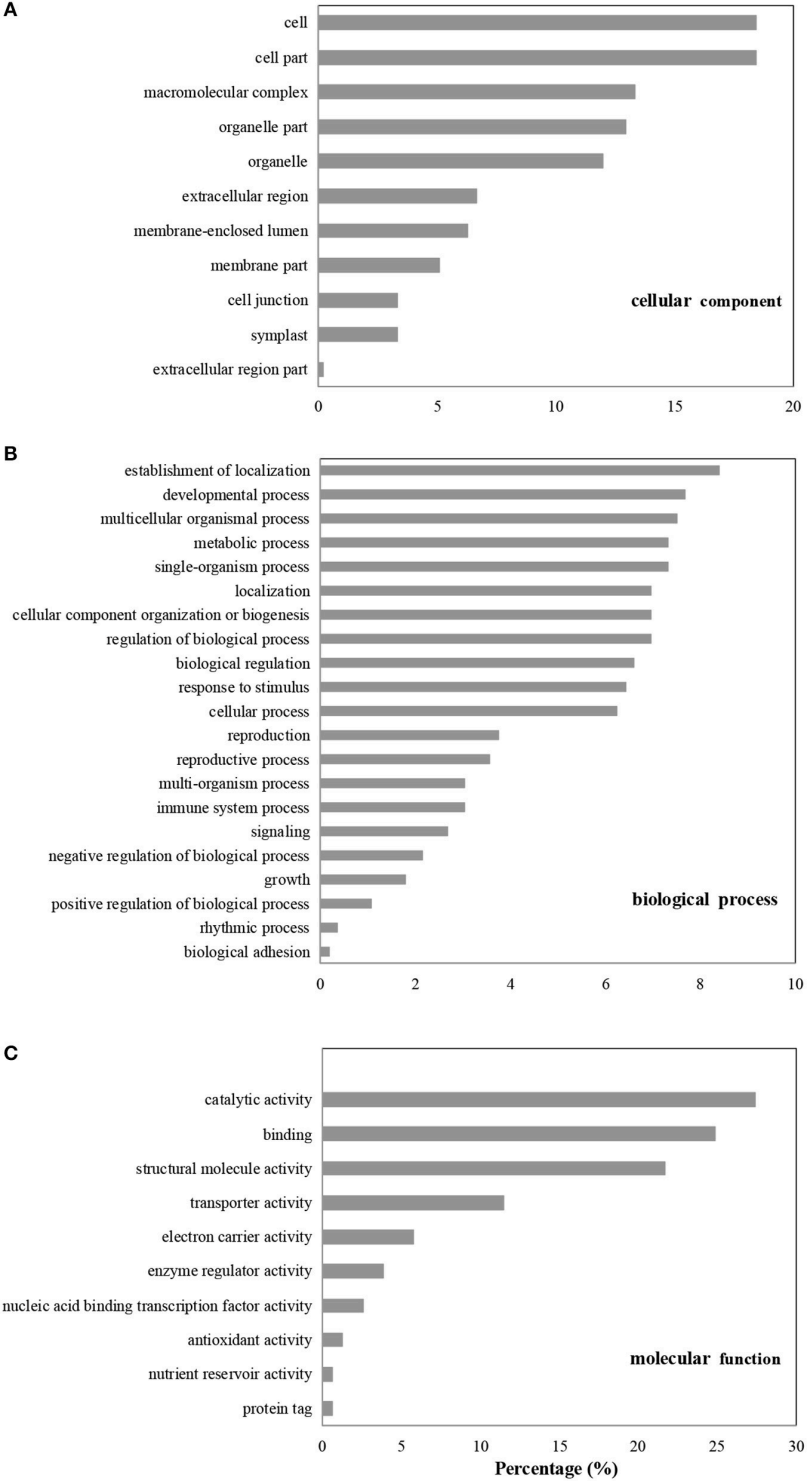
GO enrichment analysis of differentially accumulated proteins (DAPs). The DAPs were classified based on cellular component **(A)**, biological process **(B)**, and molecular function **(C)**.

A gene ontology (GO) database was used to classify the DAPs that were enriched in the *B. cinerea*-inoculated vs. the water-inoculated, control kiwifruits. Identified proteins were divided into three groups: cellular component, biological process, and molecular function. In the cellular component category, most of the enriched proteins were related to cell, macromolecular complex, and organelle (Figure 3A). In the biological process category, the most highly enriched proteins were associated with establishment of localization, as well as developmental, multicellular organismal, and metabolic processes. Other processes, such as response to stimulus and signaling, were also affected by *B. cinerea* infection (Figure 3B). In the molecular function category, the four highly enriched proteins were associated with catalytic activity, binding, structural molecule activity, and transporter activity (Figure 3C).

### KEGG and COG enrichment analysis

**Figure 4:**
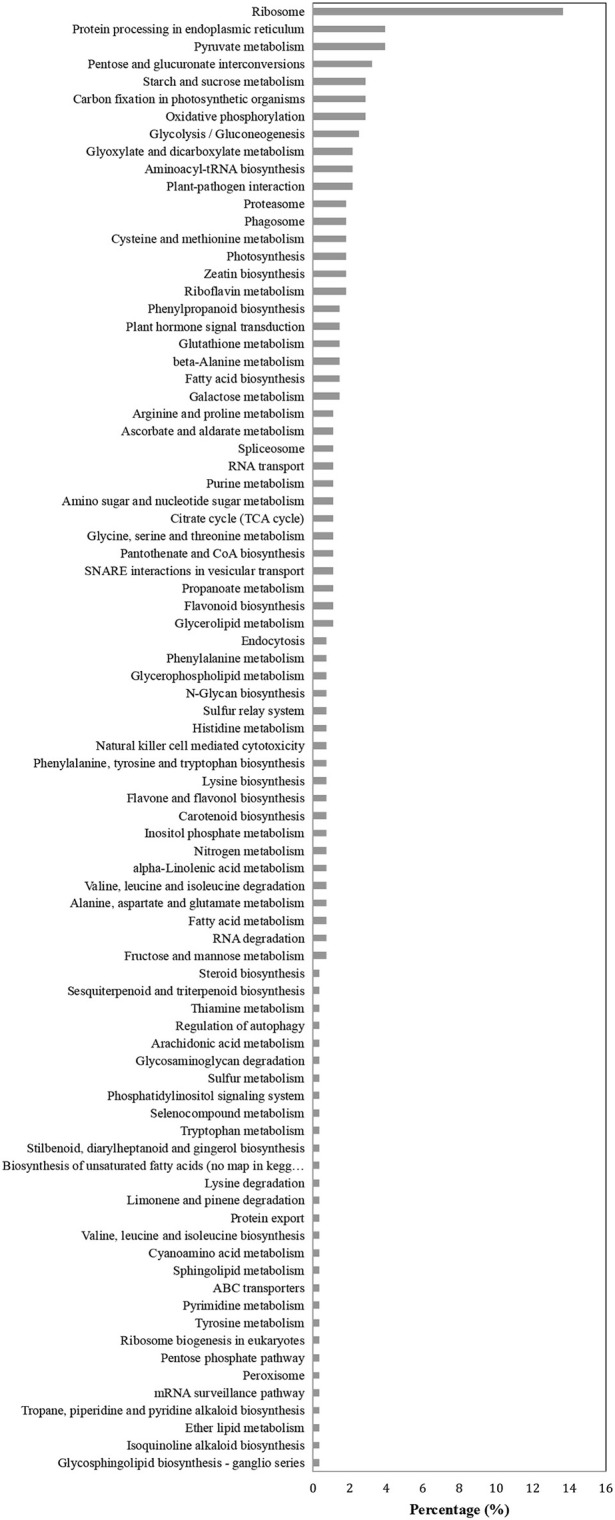
KEGG pathway enrichment analysis of differentially accumulated proteins (DAPs).

**Figure 5:**
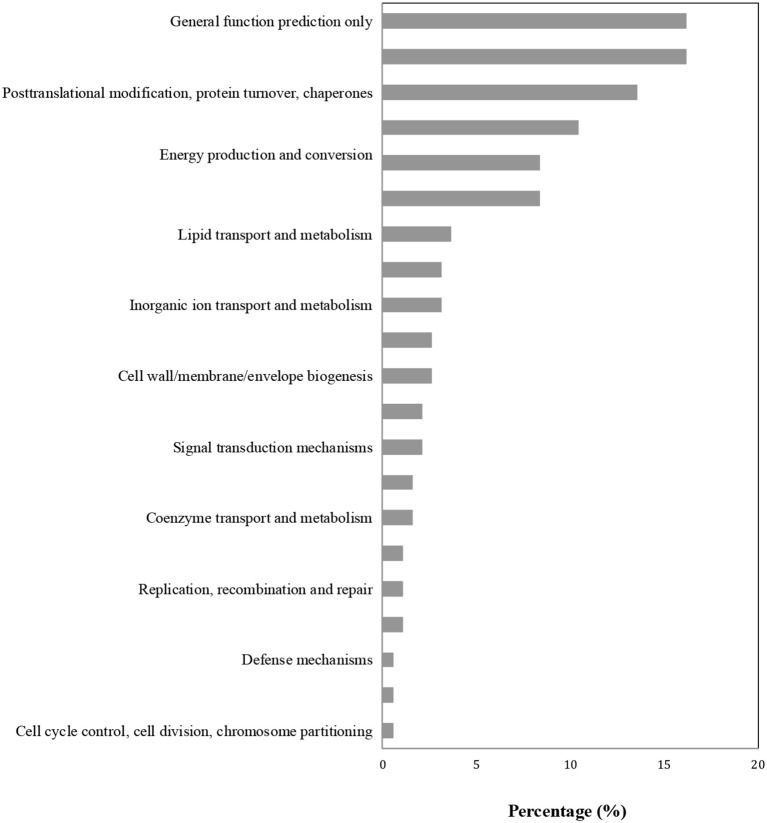
COG enrichment analysis of differentially accumulated proteins (DAPs).

Proteins in the same pathway presumably perform their biological function collectively. Pathway enrichment analysis using the KEGG database was carried out to characterize the potential biological function of the *B. cinerea*-affected proteins. As shown in Figure 4, the majority of DAPs were associated with metabolism, plant-pathogen interaction, and biosynthesis. The COG classification corresponded well with the results of the KEGG analysis. The majority of DAP proteins were associated with the categories of posttranslational modification, metabolism, signal transduction, and defense mechanisms (Figure 5).

### Penetration site reorganization and polarization

**Figure 6:**
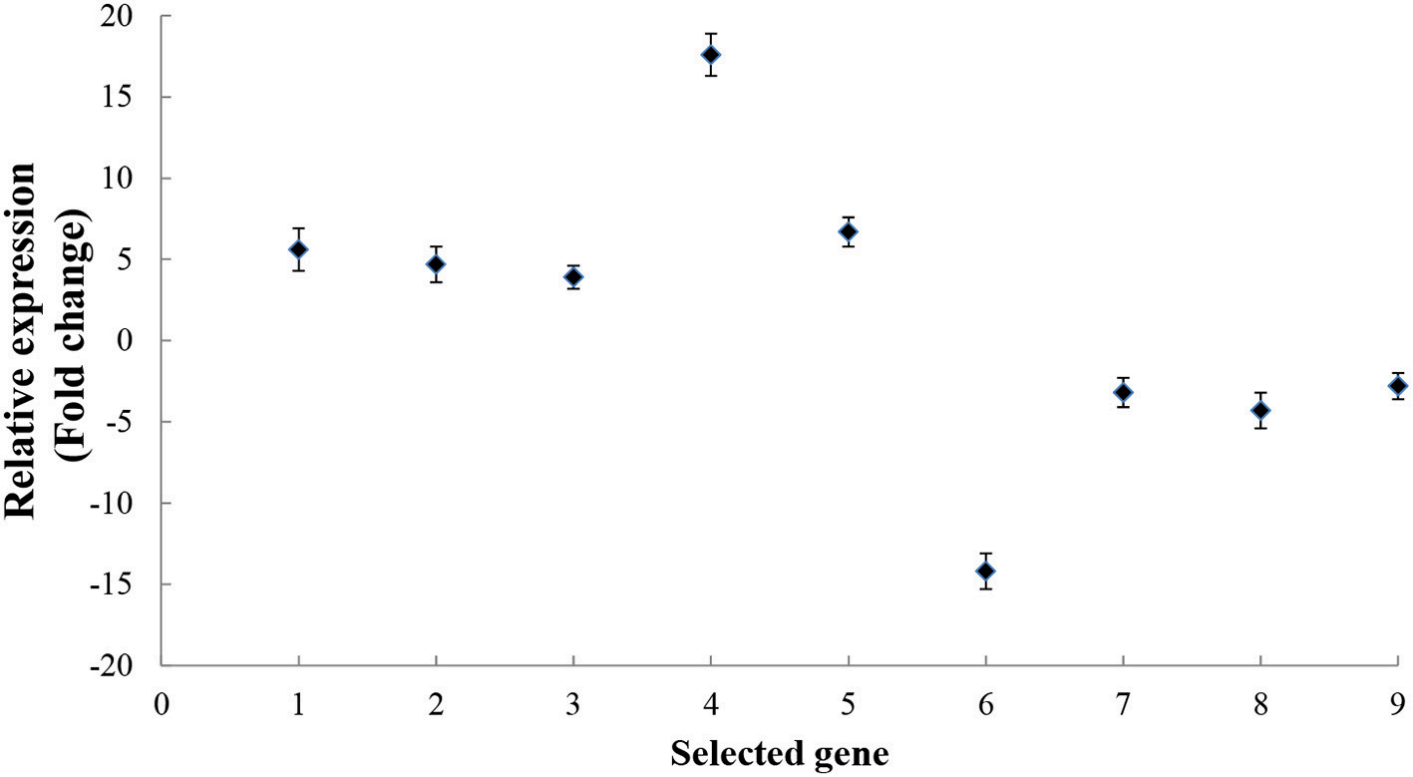
RT-qPCR analysis of kiwifruit genes encoding proteins that either increased or decreased their level of accumulation in response to *B. cinerea*. The numbers from 1 to 9 on the x axis represent the following genes in order: *Myosin 10* (Achn132881), *Pectinesterase* (Achn064441), *Polygalacturonase-inhibitor protein* (Achn126481), *Pathogenesis-related Bet v I* (Achn053521), *Alternative oxidase* (Achn228711), *Germin-like protein* (Achn331061), *Annexin* (Achn313711), *Copper/zinc superoxide dismutase* (Achn052701), and *Thaumatin* (Achn001821). Data presented are the mean ± SD of three independent experiments in which each experiment was comprised of three biological replicates for a total of *n* = 9.

Recognition is the first step in the interaction between a plant host and a pathogen. Using live-cell imaging in *Arabidopsis*, Yang et al. (2014) determined that the myosin motor protein, Myosin XI, can drive the rapid reorganization and polarization of actin filaments during the infection of *Arabidopsis* by the barley powdery mildew fungus, *Blumeria graminis* f. sp. *hordei*. In the present study, seven kiwifruit Myosin/Myosin-like proteins were identified as responding to *B. cinerea*. These included: Achn331551, Achn132881, Achn198781, Achn332471, Achn099221, and Achn115381, all of which increased in accumulation (Table 1). The expression pattern of *Achn132881* (*Myosin 10*) was also found to be up-regulated in the analysis of *B. cinerea*-inoculated kiwifruit by RT-qPCR (Figure 6).

### Characterization of *Myosin 10* function via VIGS

**Figure 7:**
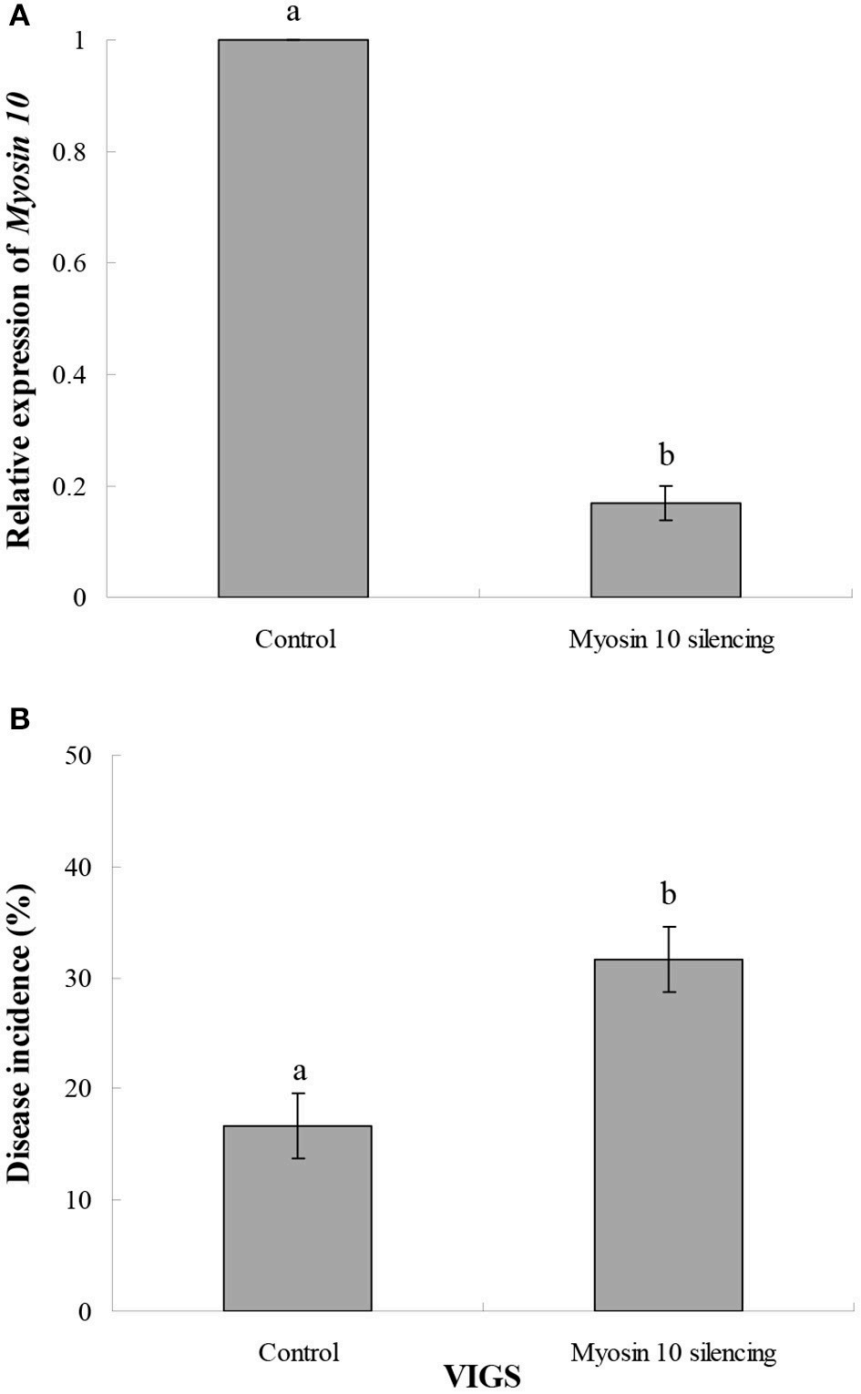
**(A)** Effect of VIGS on the relative expression of *Myosin 10* in *Myosin 10* VIGS and control kiwifruit inoculated with *B. cinerea*. **(B)** Disease incidence (%)in *Myosin 10* VIGS and control kiwifruit inoculated with *B. cinerea*. The control represents kiwifruit without *Myosin 10* silencing in which the kiwifruit was inoculated with *Agrobacterium* carrying an empty vector. Data presented are the mean ± SD of three independent experiments in which each experiment was comprised of three biological replicates for a total of *n* = 9. Column means with different letters are significantly different according to a Student's *t*-test at *P* < 0.05.

VIGS was used to characterize the function of *Myosin 10* in the infection of kiwifruit by *B. cinerea*. Results indicated that *Myosin 10* was successfully silenced by the VIGS construct (Figure 7A). Furthermore, kiwifruit in which *Myosin 10* was silenced were significantly more susceptible to *B. cinerea* than control kiwifruit based upon the analysis of disease incidence (Figure 7B). These data indicate that *Myosin 10* plays a crucial role in the defense response of kiwifruit to *B. cinerea*.

### Cell-wall degradation or reinforcement

*B. cinerea*, as a necrotrophic pathogen, initiates infection by synthesizing and secreting plant-cell-wall degrading enzymes (PCWDEs), and then delivering pathogen effectors to host cells, via specialized infection structures, that interfere with host recognition systems (Gourgues et al., 2004). On the host side, kiwifruit may initiate pathogen defense mechanisms that prevent pathogen entrance into host cells and activate other defense responses. Plant cell walls are the first defense barrier, and are rich in pectin, cellulose and hemicellulose. *B. cinerea* can invade host plants by utilizing these cell wall constituents as a nutrient source. Plants produce various proteinaceous inhibitors in order to protect themselves against microbial pathogen attack. In the present study, two putative polygalacturonase-inhibitor proteins (PGIP), Achn126481, and Achn246321, both of which contain a leucine-rich repeat (LRR), were present at significantly higher levels in inoculated tissues collected at 24 h (early infection stage) after inoculation. PGIPs are well-known to be involved in fungal pathogen resistance. Transgenic tomatoes that express a pear-fruit PGIP were shown to inhibit the growth of *B. cinerea* in ripe tomatoes (Powell et al., 2000).

The role of pectinesterases, another group of PCWDEs, is more complicated. Four putative pectinesterases, Achn064441, Achn011001, Achn064451, and Achn107321, were present in significantly higher levels in *B. cinerea*-inoculated kiwifruit at 24 h after inoculation. A proteomic analysis of tomato fruit also found that a putative pectinesterase was activated by *B. cinerea*, even during the later infection stage (3 days post-inoculation; Shah et al., 2012). Interestingly, one pectinesterase inhibitor protein, Achn317471, decreased in accumulation. Two glycoside hydrolase proteins, Achn106551 and Achn047911, also increased in accumulation. Another two glycoside hydrolase proteins, Achn235831 and Achn367241, however, decreased in accumulation. Overall, the genetic signatures in plant cell-wall-degrading enzymes seem to be affected by or drive the coevolution of plant-pathogen systems (Kubicek et al., 2014). On the one hand, a fungal pathogen needs to activate or increase hydrolase activity in order to facilitate the invasion of host tissues. On the other hand, a host plant needs to be able to inhibit hydrolase activity as a defense mechanism. A similar response pattern was observed for a glucosidase, a plant-cell-remodeling protein. Achn047911 and Achn106551, two predicted alpha-glucosidases, were both shown to accumulate to a greater level (1.37-fold) in pathogen-inoculated kiwifruit than in water-inoculated kiwifruit. In contrast, Achn235831, a predicted beta-glucosidase, exhibited a decreased level of accumulation. A previous study demonstrated that suppressing *FaBG3*, a strawberry beta-glucosidase gene, resulted in greater resistance to *B. cinerea* (Li et al., 2013). Lipases also play an important role in plant defense against pathogens in *Arabidopsis* via negative regulation of auxin signaling (Lee et al., 2009). Results in the present study revealed that Achn230831 and Achn23084, two putative lipase proteins, had lower levels of accumulation in response to *B. cinerea*. Collectively, these data suggest that they may act as negative regulators of disease resistance in kiwifruit.

### Mitogen-activated protein kinase (MAPK) cascades

MAPK cascades are highly conserved signaling modules in eukaryotes that can transduce extracellular stimuli, such as pathogen-associated molecular patterns (PAMPs) into intracellular responses (Meng and Zhang, 2013). Plant MAPK cascades play important roles in plant defense mechanisms against pathogen attack. MAPK cascades are involved in signaling multiple defense responses, such as the induction of plant defense hormones, ROS generation, defense gene activation, cell wall strengthening, and hypersensitive response (Jalmi and Sinha, 2016; Lee and Back, 2017).

Ras proteins can activate MAPK cascades (Kawano et al., 2010). In our study, Achn389291, a putative Ras-related Rab-2-A protein, had higher levels of accumulation in pathogen-inoculated kiwifruit. Pathogens, however, can utilize effectors to suppress plant MAPK activation and downstream defense responses in order to promote pathogenesis. The level of Achn008501, a predicted small GTPase ADP ribosylation factor, decreased by 0.69-fold in response to *B. cinerea* infection. This finding is consistent with a previous study (Takác et al., 2013), in which wortmannin, a MAPK (PI3K) inhibitor, decreased the level of the vacuolar trafficking protein RabA1d, a small GTPase that regulates vesicular trafficking in the trans-Golgi network. Another study revealed that a small GTPase ADP ribosylation factor 6 (ARF6) and its effector phospholipase D2 (PLD2) interfere with exosomes by controlling the budding of intraluminal vesicles into multivesicular bodies (MVBs) (Ghossoub et al., 2014). In our study of kiwifruit, Achn061151, a predicted charged MVB protein 4b, exhibited higher levels in response to *B. cinerea*. Wang et al. (2014) reported that LYST-interacting protein 5 (LIP5) in *Arabidopsis* could be activated by MPK3 and MPK6 MAPK cascades. LYST-interacting proteins induce the membrane dissociation of endosomal sorting complexes required for transport proteins or MVB synthesis. Further functional studies will be required to elucidate the role of Achn061151 in the response of kiwifruit to *B. cinerea*.

### Ubiquitin-26S proteasome system

The ubiquitin-26S proteasome system (UPS) plays an important role in various signal transduction pathways by controlling the abundance of key regulatory proteins and enzymes. Achn197261 and Achn314841, two predicted proteasome subunit alpha type proteins, exhibited increased levels of accumulation in response to *B. cinerea* at 24 h post-inoculation. Similar results were reported by Pan et al. (2013), who found that a proteasome subunit alpha type protein was induced in tomato fruit by the necrotrophic fungal pathogen, *Rhizopus nigricans*, at 48 h post-inoculation. Achn369161 and Achn022881, two predicted proteasome subunit beta type proteins, however, exhibited decreased levels in response to infection. Additionally, Achn136801, a predicted 26S proteasome non-ATPase regulatory subunit, also exhibited a significantly decreased level of accumulation. Thus, the underlying function of these proteins appears to be complex. On one hand, a host plant can potentially defend itself from pathogen attack by activating the UPS to trigger a hypersensitive response, leading to programmed cell death (PCD) at the infection site (Kachroo and Robin, 2013). On the other hand, a pathogen may attempt to suppress immunity-associated PCD or manipulate the host UPS to inhibit host defense proteins and/or enzyme activity (Janjusevic et al., 2006).

### Pathogenesis-related (PR) proteins

PR proteins can be grouped into several classes based on the organization of specific amino acid motifs and membrane-spanning domains, two of which are a LRR domain and a START-like domain protein. Results of the present study revealed that two likely LRR proteins, Achn126481 and Achn291371 exhibited increased levels in response to inoculation with *B. cinerea*. The role of LRR proteins in disease resistance has recently been well documented. In a transcriptomic analysis, LRR genes, such as *RGA2* or *FEI1*, in faba bean (*Vicia faba* L.) have been reported to be involved in resistance to *Ascochyta fabae* infection (Ocaña et al., 2015). Park et al. (2012) found that over-expression of rice LRR protein resulted in the activation of a defense response, thereby enhancing resistance to bacterial soft rot in Chinese cabbage, while Wang et al. (2016), using overexpression and gene silencing approaches, reported that the wheat homolog of the nucleotide-binding site-LRR resistance gene, *TaRGA*, contributed to resistance against powdery mildew (*B. graminis*). Achn053521, a predicted major latex-like protein that possesses a START-like domain, also increased in accumulation in response to *B. cinerea* infection in the present study. Gai et al. (2017) reported that when the latex protein *HMLX56* from mulberry (*Morus multicaulis*) was ectopically expressed in Arabidopsis, the transgenic plants showed enhanced resistance to *B. cinerea* and the bacterial pathogen *P. syringae* pv. tomato DC3000. Thaumatin-like proteins (TLPs), PR protein family members, can inhibit fungal pathogen growth. Certain TLPs have been found to be associated with stress response, such as the heat shock response (Durand et al., 2012). In the present study, Achn001821, a predicted TLP, exhibited decreased levels in response to *B. cinerea* at 24 h post-inoculation. In contrast, a TLP in “Amarone” wine grapes was induced by *Penicillium expansum* in response to water stress (Lorenzini et al., 2016). This finding indicates that DAPs may have different roles in response to abiotic and biotic stresses.

### Transcription factors

The heat-shock factor-like transcription factor BF1 functions as a major molecular switch in the transition from plant growth to plant defense (Pajerowska-Mukhtar et al., 2012). Our results identified seven predicted heat shock proteins, Achn092681, Achn089541, Achn001561, Achn079561, Achn383281, Achn011721, and Achn071381, that increased in their accumulation in response to *B. cinerea*. WD-repeat-domain-related transcription factors have been demonstrated to play an important role in jasmonate (JA) signaling (Qi et al., 2014). JAs are a class of lipid-derived hormones that regulate various defense responses against pathogens and insects (Wasternack and Hause, 2013; Zhang et al., 2017a). Perception of a pathogen or insect invasion induces the synthesis of jasmonoyl-L-isoleucine (JA-Ile), which binds to the COI1-JAZ receptor, triggering the degradation of JAZ repressors and activates transcriptional reprogramming associated with plant defense (Zhang et al., 2017b). In our study, two predicted WD-repeat proteins, Achn228601 and Achn388771, exhibited increased levels of accumulation in response to *B. cinerea*.

### ROS signaling pathway

The ROS signaling pathway plays an important role in plant immunity. Oxidative bursts can trigger pathogen resistance responses (Camejo et al., 2016). Our results indicate that the accumulated level of a predicted glutathione S-transferase, Achn144051, increased in kiwifruit in response to infection by *B. cinerea*, however, another predicted glutathione S-transferase, Achn259181, decreased. This indicates that various glutathione S-transferases respond differently to the presence of a pathogen. Similar results were observed in grapevine (*Vitis vinifera* cv. Gamay) cells by Martinez-Esteso et al. (2011). In their comparative proteomic study, two grape peroxidases increased in response to methyl jasmonate, while two decreased. In addition, Achn296481 (a predicted sulfur transferase), Achn147891 (a predicted cysteine desulfurase), Achn052701 (a predicted superoxide dismutase), and Achn285991 (a predicted peroxidase) all exhibited decreased levels of accumulation in response to *B. cinerea*.

### Other proteins

The elemental defense hypothesis assumes that the hyper-accumulation of heavy metals, such as zinc, nickel, or cadmium, in their tissues can protect host plants from pathogen attack. In the present study, a heavy-metal-associated protein, Achn358621, increased in response to *B. cinerea*. A previous proteomic study in rice reported that enzymes involved in the Calvin cycle and glycolysis decreased in response to infection by the fungus, *Cochliobolus miyabeanus* (Kim et al., 2014). In our study, the level of seven predicted glycolysis-related proteins, Achn305831, Achn364961, Achn239461, Achn349661, Achn114051, Achn310551, and Achn350451 were also observed to decrease in response to infection. Some unknown proteins, with potential functions based on GO annotation, are worthwhile to be further investigated. For example, Achn277891 involved in abiotic stress response (GO: 0009651) may also participate to the response of kiwifruit to the biotic stress caused by *B. cinerea*; while Achn095331 involved in oxidation-reduction process (GO: 0055114) may play a role in the ROS signaling pathway.

### RT-qPCR analysis

Nine genes coding for proteins that either increased or decreased their level of accumulation in response to *B. cinerea* in the proteomic analysis were selected for RT-qPCR analysis, in order to determine whether or not the DAPs were also up- or down-regulated at the transcriptional level. Results indicated that the expression level of all nine of the selected genes exhibited a pattern of expression (Figure 6) similar to the pattern of accumulation exhibited by their respective proteins in the proteomic analysis (Table 1).

## Conclusions

The present study provides new insight into the interaction that occurs between kiwifruit and *B. cinerea* during the infection process. A set of DAPs of kiwifruit associated with penetration site reorganization, cell wall degradation, MAPK cascades, ROS signaling, and PR proteins were identified. Using VIGS, *Myosin 10* was shown to play a crucial role in modulating resistance to host penetration by *B. cinerea*. The information from this study may contribute to the development of new approaches and management methods for the effective control of gray mold in kiwifruit.

## Author contributions

YS and YoL: conceived and designed the experiments; JL, YoL, and HC: performed the experiments; JL and YiL: analyzed the data; JL, YS, and YoL: drafted the manuscript; YiL: revised the manuscript critically. All authors read and approved the final manuscript.

### Conflict of interest statement

The authors declare that the research was conducted in the absence of any commercial or financial relationships that could be construed as a potential conflict of interest.

## Supplementary material

Figure S1 A workflow diagram of the iTRAQ-based quantitative proteomic analysis.

Table S1 Gene-specific primers used in the RT-qPCR analysis.

Table S2 List of the 2,487 proteins identified by LC-ESI-MS/MS using iTRAQ.
