# Supplementary figures and images for: Proteomic Analysis of Kiwifruit in Response to the Postharvest Pathogen, Botrytis cinerea
Source: Front Plant Sci. 2018 Feb 15;9:158. doi: 10.3389/fpls.2018.00158 (PMC5818428; doi:10.3389/fpls.2018.00158)

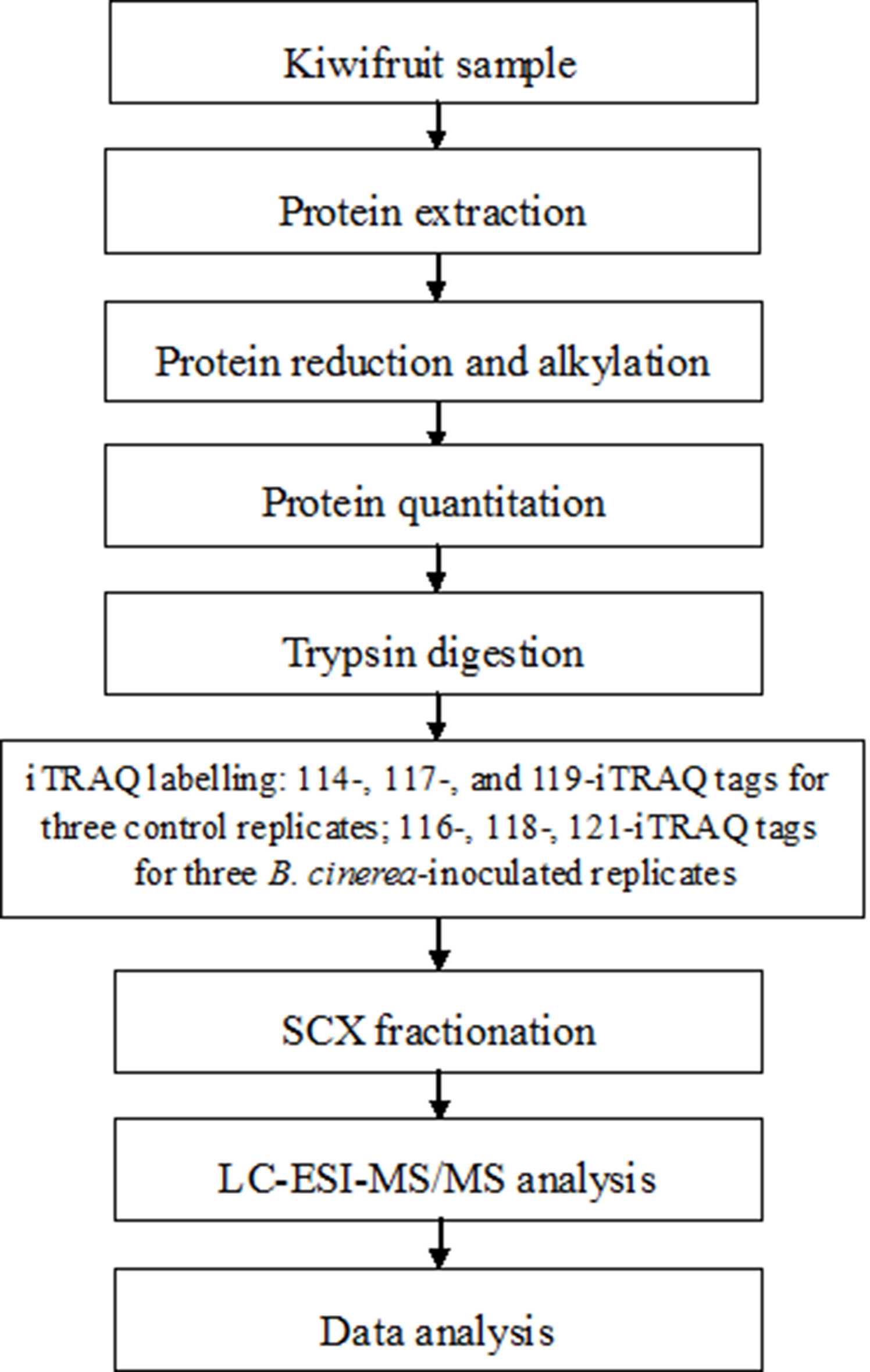

Supplement: Figure S1 — A workflow diagram of the iTRAQ-based quantitative proteomic analysis. [file Image1.TIF]
